# Inconsistency between Self-Reported Energy Intake and Body Mass Index among Urban, African-American Children

**DOI:** 10.1371/journal.pone.0168303

**Published:** 2016-12-15

**Authors:** Miwa Yamaguchi, Elizabeth Anderson Steeves, Cara Shipley, Laura C. Hopkins, Lawrence J. Cheskin, Joel Gittelsohn

**Affiliations:** 1 Department of Nutritional Science, National Institute of Health and Nutrition, National Institutes of Biomedical Innovation, Health and Nutrition, Tokyo, Japan; 2 Department of International Health, Global Obesity Prevention at Johns Hopkins, Johns Hopkins Bloomberg School of Public Health, Baltimore, Maryland, United States of America; Pennington Biomedical Research Center, UNITED STATES

## Abstract

**Background:**

To prevent obesity, it is important to assess dietary habits through self-reported energy intake (EI) in children. We investigated how EI is associated with body mass index and which elements of dietary habits and status are associated with EI among African-American (AA) children.

**Methods:**

We assessed and included data from 218 10–14-year-old AA children in Baltimore, MD, USA. EI was calculated using a food frequency questionnaire. The basal metabolic rate (BMR) was used as the predicted minimal rate of energy expenditure of children. A fully adjusted multiple logistic regression was used to determine the prevalence of obesity (≥ 95^th^ BMI-for-age percentile) among the quartiles of EI/BMR ratio using the third quartile for the reference. The differences in the age-adjusted mean EI/BMR among the categories of dietary habits, social support, and socio economic status were analyzed using a general linear model.

**Results:**

Children with the lowest EI/BMR had significantly higher adjusted odds ratio (aOR) of obesity as compared to those in the third quartile of EI/BMR (boys aOR 4.3; 95% confidence interval 1.08, 20 and girls aOR 4.1; 1.02, 21). In girls, the adjusted mean EI/BMR in the group that prepared food less than the means (3.8 times/week) was significantly lower than the group that prepared food over the means (*P* = 0.03). Further, the group that reported eating breakfast under 4 times/week indicated an adjusted mean EI/BMR lower than the group that ate breakfast over 5 times/week in both sexes.

**Conclusions:**

When EI was under-reported with reference to BMR, we may observe high prevalence of obesity. Further, food preparation by children and frequent consumption of breakfast may instill food cognition with usual dietary habits. Therefore, holistic assessments including dietary habits are required to examine self-reported food intake especially among overweight/obese children.

## Introduction

School children are increasingly choosing and consuming foods on their own outside their home [1]. Therefore, the quality and quantity of foods children consume needs to be assessed through their self-report, especially because their caregivers and other adults cannot provide adequate information on this topic. Self-reported dietary intake is widely used for investigating food-related indicators in public health. There are several processes that play a role in the cognitive process of self-reporting of children’s diet, including the children’s attention, interpretation, food label information, retention, retrieval, and response formulation regarding food [1, 2]. Unhealthy eating habits, such as snacking frequently, makes it difficult for children to pay attention to frequency of food consumption and portion sizes. It has been reported that increasing energy consumption, unstructured eating patterns, and frequency of out-of-home eating may lead to loss of motivation and forgetfulness, resulting in the misreporting of dietary habits [2]. In adolescence, social desirability concerning of body shape and body image may exaggerate these factors, which may impact food consumption behaviors [1, 2]. However, the relationship between misreporting of food intake and body mass index, and the bias of self-reported food intake assessment is still unclear.

According to the Youth Risk Behavior Surveillance System survey in 2013, which used self-reported height and weight assessments, 17.0% of 14-18-year-old African-American (AA) youth residing in Baltimore City, Maryland, were obese [3]. Our previous data showed that 43% of AA children aged 10–14 years were overweight or obese [4]. We implemented the *B’More Healthy Communities for Kids* (*BHCK*) trial, a multi-level and systems-based childhood obesity prevention strategy for low-income children (predominantly AA children) aged 10–14 years, to develop and evaluate a community-based obesity prevention program in Baltimore [5]. The objective of the present study was not to evaluate the intervention of the *BHCK* project, but to report data from its wave1 baseline sample. We aimed to investigate whether self-reported food intake is associated with overweight/obesity among AA children in low-income, urban neighborhoods. Furthermore, to understand the background of the self-reported food intake of the sample, we investigated its association with their dietary habits, social support for food and physical activity habits, and socioeconomic status [6].

## Methods

### Participants

Participants from the *BHCK* wave 1 trial were recruited from 14 neighborhood zones around recreation centers in low-income, predominantly AA neighborhoods in Baltimore. The participants were recruited from a variety of venues and events, such as recreation centers, libraries, swimming pools, grocery stores, and back-to-school events. We also included other means of recruitment, such as handing out fliers to the neighborhoods on streets and in stores, and engaging children and members of the community in conversations. Eligible participants were chosen based on three criteria. First, the subject had to be aged 10 to 14 years at the time of recruitment. Children ages 10-14-year-old express increasing autonomy around food choice and decision-making. The data in this current study are drawn from the baseline sample in implementing an intervention trial targeting AA adolescents [5]. Second, the participant had to live within a 1.5-mile radius of the recreation center in that neighborhood zone. Third, self-report of the participants that they did not intend to relocate within the next two years. For each neighborhood, recruitment was conducted until at least 75 eligible subjects and interested youth-parent dyads were recruited. Then, a sampling frame was created for each zone, and 24 youth-caregiver dyads were randomly selected for participation from each zone [5, 7].

We enrolled 283 eligible adolescents (boys: n = 131, girls: n = 152) in the present study. During analysis, we excluded individuals who had provided no or unclear information about weight, height, and date of birth, which were necessary for the calculation of the BMI-for-age percentile (boys: n = 1, girls: n = 4). Furthermore, an exclusion criterion of implausible dietary intake was set at < 500 kcal or no data of energy intake. Thus, 2 boys and 5 girls were excluded from participation on these grounds [8, 9]. The children who had no information of the frequency of breakfast consumption were also excluded (boys: n = 13, girls: n = 26). After excluding the participants with no information regarding the frequency of food preparation by a household member and children, the caregiver’s education level, and annual household income, the primary outcomes were assessed in a final sample of 218 children (boys: n = 109, girls: n = 109).

Written assent from the children and written consent from their next of kin, caretakers, or guardians, were obtained during data collection. The Institutional Review Board of the Johns Hopkins Bloomberg School of Public Health approved the study.

### Anthropometric data

Anthropometric data (height and weight) were collected from each child, using a Seca 213 Portable Measuring Rod stadiometer and Tanita BF697W Duo Scale. One trained interviewer measured the body weight and height of the children, who were wearing light clothing. In very rare cases when the hairstyle impeded measurement, the child was asked to adjust the hairstyle to allow for a more accurate measure. Each measure was taken in duplicate and repeated third time if the first two differed by more than 0.64 cm (0.25 inch) or 0.09 kg (0.2 lbs). The mean of the two or three measures was used to indicate weight or height. BMI was calculated as body weight (kg) divided by the square of height (m). BMI-for-age percentile was calculated with the Excel BMI calculator, English version, based on 2000 Centers of Disease Control and Prevention (CDC) growth charts [10, 11]. Overweight was defined as exceeding the 85^th^ BMI-for-age percentile, while obesity was defined as exceeding the 95^th^ BMI-for-age percentile.

### Self-reported energy intake using a food frequency questionnaire

Self-reported energy intake (EI) was calculated by the *Block Kids 2004 Food Frequency Questionnaire* (*Block Kids FFQ*), which is an FFQ (Nutrition Quest, Berkley, CA) validated in youth ages 10–17, that solicits information about the frequency and amount of 77 food/beverage items consumed within the past seven days [12, 13]. The same interviewer read out all the questions of the *Block Kids FFQ* to the interviewee, and recorded children’s responses. During the interview with a child, his/her parents or family members were separated as far as the space permitted, so that they would not hear the children’s responses to questions that may be influenced by parental presence (i.e., questions that asked about parent/child interactions around food). Interviewers administered the *Block Kids FFQ* for children’s dietary data, however, if a child had a specific question about a food they had consumed at home that was to be reported on the FFQ, they were permitted to ask a parent. To assist the interviewees into quantifying portion size, a trained interviewer provided the interviewee with portion estimation aids comprising representative amounts of generic foods in standard serving sizes on plates and bowls or photographed pictures of these representations. The children’s self-reported consumption of the food/beverage items was classified according to the following six categories, indicating the frequency of food intake: none = 0.1 (times/week), 1 day = 1, 2 days = 2, 3–4 days = 3.5, 5–6 days = 5.5, and every day = 7. Five questions about specific varieties of food items, such as low-fat milk, whole milk, and the usual size of soda containers provided further detail for some of the responses (e.g., 12 oz. can, 20 oz. bottle). The quantity the child consumed was assessed through 3–4 response categories related to the type of food. The consumption of the amount of whole-wheat and white bread was reported in terms of slices (e.g., 1, 2, 3–4, 5 or more), and items such as chicken nuggets were reported in terms of the number of pieces (e.g., 1–4). The values recorded in the nutrient/food group database for each food item on the questionnaire were population-weighted average-intake values per 100 g (0.2 lbs) of food for calculation of total energy intake according to the *Block Kids FFQ* [14]. The percent energy from micronutrients (protein, fat, and carbohydrate) was used to adjust EI. To investigate the consumption of confectionaries, we used two food items; sweets and desserts (% energy) and sugary beverages (g/day) in the *Block Kids FFQ*. The ratio of sugary beverages with EI were used to adjust EI (g/EI). These food items and nutrient database values were based on data from the National Health and Nutrition Examination Survey (NHANES) 1998–2002 and the NHANES 2003–2004 [15].

### Prediction of basal metabolic rate (BMR)

BMR is the minimal rate of energy expenditure compatible with life, which was calculated by the equation generated by the Food and Agriculture Organization/World Health Organization/United Nations University (FAO/WHO/UNU) among 10–18-year-old boys and girls [16]. We indicate the equation of BMR below:

Boys:16.6 * weight (kg)+77 * height (m)+572=BMR

Girls:7.4 * weight (kg)+482 * height (m)+217=BMR

After calculating BMR for all the children, EI was divided by BMR to indicate the ratio of difference in energy intake (EI/BMR). The EI/BMR ratio was stratified into quartiles, as follows: first quartile: ≤ the 25^th^ percentile; second quartile: the 25^th^ percentile–50^th^ percentile; third quartile: the 50^th^ percentile–75^th^ percentile; and fourth quartile: > the 75^th^ percentile.

### Child Impact Questionnaire (CIQ) and Adult Impact Questionnaire (AIQ)

The same trained interviewer read the questions on the *CIQ* to the child interviewee, and recorded his/her responses on the questionnaire. A second trained interviewer completed the *AIQ* with the child’s parent or caregiver. The *CIQ* and *AIQ* pertained to demographics and frequency of food preparation by the child and a household member, respectively [5]. The frequency of food preparation by children or by a household member, within the last seven days, was classified into the following six categories: never = 0, once/week = 1, 2–3 times/week = 2.5, 4–6 times/week = 5, once/day = 7, and 2 or more times/day = 14. To investigate the social support level for food and physical activity habits among children, we used the following 7 original items from the *CIQ*: “Is there someone in your life who… 1) Talks to you about making improvements in your food and physical activity habits, 2) encourages you to keep making healthy choices even when you don’t feel like it, 3) shows you how to make healthy choices by setting a good example, 4) praises you about making changes in your diet and physical activity habits, 5) will be your buddy with making food and physical activity changes together, 6) helps you solve problems that get in the way of your eating healthy and being active, and 7) tells you about new healthy foods and encourages you to try new healthy foods [7]. A “yes” response to each question scored 1, while a “no” response was scored 0. The relevant internal consistency of the social support score was indicated by the Cronbach’s alpha (α = 0.72).

We used caregiver’s self-reported education level and annual household income as measures of socioeconomic status. These were self-reported by the children’s caregivers on the *AIQ*. The highest degree of the caregiver’s education level was classified into two categories: up through high school level, including a General Education Development (GED) or less and over college level; the individuals who completed at least some colleges or higher. The annual household income (US dollars) was combined into three categories among the nine groups used in previous reports: ≤ 20,000, > 20,000 − ≤ 40,000, and > 40,000 [17].

### Statistical analysis

Due to the skewed distribution, continuous variables were log-transformed. The exponentiated value of the log-transformed was indicated in the result. Continuous variables were analyzed by a student t-test for assessing sex differences, except for the social-support score (unit), which was analyzed using Wilcoxon rank-sum test. Chi-square test was used to analyze the proportional sex differences in the categorical variables such as overweight or obese status, caregiver’s education level (under high school level, over college level) and annual household income (≤ 20,000, > 20,000 − ≤ 40,000, and > 40,000 US dollars). One-way analysis of variance (one-way ANOVA) was performed to analyze the differences in the EI/BMR quartiles on micronutrients and food items. The mean of BMI-percentile-for-age, micronutrient variables, and food items in each quartile of EI/BMR were analyzed by the Dunnett’s test to compare these with the third quartile of EI/BMR. The age-adjusted mean of EI/BMR in each item of dietary habits, social support, and socioeconomic status were analyzed using analysis of covariance (PROC GLM), including the frequency of food preparation by a household member and by children (categorized by mean), frequency of breakfast (0–2 times/week, 3–4 times/week, and ≥ 5 times/week) [18, 19], caregiver’s education level, annual household income, and social-support score (categorized by mean). A Tukey’s test was used for comparing the adjusted mean of EI/BMR across the three categories of frequency of breakfast. Further, age-adjusted odds ratio (aOR) of overweight and obesity were analyzed by multiple logistic regression models (PROC LOGISTIC) in each quartile of EI/BMR, to compare them with the third quartile as a reference (Model 1). Frequency of breakfast was used for further adjustment in the model (Model 2). In addition to Model 2, frequency of food preparation by children (times/week), frequency of breakfast (times/week), social support score (unit), caregiver’s education level (under high school level and over college level), annual household income (≤ 20,000, > 20,000 − ≤ 40,000, > 40,000) were added in Model3. Trend-testing of the quartile of EI/BMR was undertaken using ordinal categorical variables (1, 2, 3, and 4) in an age-adjusted multiple logistic regression analysis. The criteria of significance was set at α = 0.05, and the Bonferroni correction (α = 0.05/8) was used for a multiple-comparison correction in each sex. The statistical power (1-*β*) was 0.7 when the effect size was 0.3, the probability of α error was 0.05, the sample size was 109, and the number of group was 4 using one-way ANOVA. We used the SAS system (version 9.3 SAS Institute Inc.) for statistical analysis.

## Results

Table 1 shows the sex differences in the participants’ baseline demographics. The geometric means (GM) of EI (boys: 1656 kcal/day GM ± geometric standard deviation (GSD) = 953, 2879; girls: 1559 kcal/day GM ± GSD = 894, 2719) and ratio of EI/BMR (boys: 1.06, GM ± GSD = 0.58, 1.96; girls: 1.2, GM ± GSD = 0.65, 2.05) did not show a significant difference between boys and girls. However, the mean of BMR in boys was significantly higher than that in girls (boys: 1557 kcal_th_ GM ± GSD = 1294, 1873; girls: 1350 kcal_th_ GM ± GSD = 1195, 1526). There was a significant difference between the sexes on the proportion of caregiver’s education level, annual household income, and social-support score.

**Table 1:** Sex Differences in the Baseline Demographics of the Study Participants.

	Boys		Girls			
	n (%)	GM (GM ± GSD)	n (%)	GM (GM ± GSD)	*P*	
**Age (years)**						
10–11	46 (42.2)		53 (48.6)		0.616	N.S.
12–13	43 (39.5)		37 (34.0)			
14	20 (18.5)		19 (17.4)			
**Body composition**						
Body weight (kg)	109	51.0 (36.9, 70.3)	109	51.9 (37.6, 71.6)	0.690	N.S.
BMI-percentile-for-age (%)	109	59.4 (33.6, 105)	109	61.9 (29.8, 129)	0.643	N.S.
Overweight	43 (39.5)		49 (45.0)		0.411	N.S.
Obesity	22 (20.2)		24 (22.0)		0.740	N.S.
**Energy- intake and—expenditure**						
Self-reported energy intake: EI (kcal/day)	109	1656 (953, 2879)	109	1559 (894, 2719)	0.423	N.S.
Basal metabolic rate: BMR (kcal_th_)	109	1557 (1294, 1873)	109	1350 (1195, 1526)	<0.001	**
EI/BMR	109	1.06 (0.58, 1.96)	109	1.2 (0.65, 2.05)	0.310	N.S.
**Dietary habit**						
Frequency of food preparation (times/week)						
A household member	109	5.6 (2.4, 12.0)	109	6.2 (2.7, 12.8)	0.369	N.S.
Children	109	3.5 (0.9, 9.8)	109	3.8 (1.0, 10.1)	0.658	N.S.
Frequency of breakfast (times/week)	109	3.7 (3.1, 7.09)	109	3.2 (2.6, 6.7)	0.074	N.S.
0 − 2	19 (17.4)		27 (24.8)		0.307	N.S.
3 − 4	30 (27.5)		23 (29.4)			
≥ 5	60 (55.1)		59 (45.9)			
**Social support**						
Social-support score (units) ^a^	109	6.0 (5.0, 7.0)	109	7.0 (6.0, 7.0)	0.006	*
**Socioeconomic status**						
Caregiver’s education level						
Under high school level	58 (53.2)		76 (69.7)		0.012	*
Over college level	51 (46.8)		33 (30.3)			
Annual household income						
≤ 20,000 (US dollars)	45 (41.3)		64 (58.7)		0.032	*
> 20,000 − ≤ 40,000	47 (43.1)		35 (32.1)			
> 40,000	17 (15.6)		10 (9.2)			

Sex difference was assessed by student t-test (continuous variables), Wilcoxon rank-sum test (social-support scores), and chi-square test (categorical variables). GM: geometric mean, the exponentiated value of the log-transformed mean. GSD: geometric standard deviation, the exponentiated value of the standard deviation of the log-transformed value. N.S.: not significant

^a^The result of social-support score indicated median (25^th^ percentile, 75^th^ percentile).

*P < 0.05,

** *P* < 0.001

To indicate the consistency between quartiles of EI/BMR and body composition, Table 2 shows the differences in age, BMI-percentile-for-age, micronutrients, and confectionaries (sweetened beverages and desserts) by quartiles of EI/BMR. BMI-percentile-for-age showed a significant difference across the quartiles of EI/BMR in boys (*P* = 0.001) but not in girls (*P* = 0.665). Further, in boys, the mean of the BMI-percentile-for-age in the lowest quartile of EI/BMR was significantly higher than that in the third quartile of EI/BMR (*P* < 0.05). The three micronutrients did not show significant differences between the sexes. However, there was a significant difference in the consumption of sugary beverages between the quartiles of EI/BMR in boys and girls (*P* < 0.001). As compared with the sugary beverages consumed by those in the third quartile, the other quartiles showed significantly lower means in boys (*P* < 0.005) but not significant in girls (*P* = 0.055). However, the ratio of consumption of sugary beverages in EI did not show a significant association between the quartiles of EI/BMR in both the sexes. Energy percent from sweets and dessert did not show a significant difference among the quartiles in both sexes.

**Table 2:** The Differences in Age, BMI-Percentile-For-Age and Dietary Intake Across the Quartiles of EI/BMR.

	EI/BMR					
	First quartile (under-reporting)	Second quartile	Third quartile	Fourth quartile (over-reporting)	*P*^a^	
**Boys: EI/BMR**	**< 0.65**	**≥ 0.65 and <1.00**	**≥ 1.00 and < 1.68**	**≥ 1.68**		
**n (%)**	27 (24.8)	27 (24.8)	28 (25.7)	27 (25.7)	-	
**EI (kcal/day)**	882 (729, 1069)	1256 (1030, 1533)	2051 (1606, 2619)	3283 (2374, 4540)	-	
**Age (years)**	12.2 (10.9, 13.7)	11.7 (10.4, 13.1)	12.1 (10.8, 13.6)	11.3 (10.1, 12.8)	0.094	N.S.
**BMI-percentile-for-age (%)**	85.2 (70.1, 104) ^¶^	55.3 (31.0, 98.7)	54.2 (26.9, 109)	49.0 (28.6, 83.9)	0.001	**
**Nutritional intake**						
Protein (% energy)	13.3 (11.0, 16.1)	13.0 (10.5, 16.0)	13.0 (11.1, 15.2)	13.0 (11.3, 14.9)	0.955	N.S.
Fat (% energy)	32.6 (26.8, 39.5)	32.1 (27.7, 37.2)	32.9 (28.9, 37.4)	34.9 (30.3, 40.1)	0.223	N.S.
Carbohydrate (% energy)	54.6 (47.5, 62.7)	55.5 (50.1, 61.5)	55.1 (49.6, 61.6)	53.3 (47.5, 59.9)	0.621	N.S.
**Intake of confectionaries**						
Sweets and desserts (% energy)	11.9 (4.6, 28.6)	14.6 (7.8, 26.8)	13.7 (7.7, 23.7)	13.9 (8.0, 23.5)	0.708	N.S.
Sugary beverages (g/day)	74.1 (9.8, 520) ^¶^	152 (28.6, 795)	294 (109, 793)	405 (107, 1521)	< 0.001	**
Sugary beverages (g/EI)	0.11 (0.03, 0.41)	0.15 (0.04, 0.44)	0.15 (0.06, 0.34)	0.14 (0.06, 0.30)	0.783	N.S.
**Girls: EI/BMR**	**< 0.74**	**≥ 0.74 and <1.13**	**≥ 1.13 and < 1.64**	**≥ 1.64**		
**n (%)**	28 (25.7)	27 (24.8)	27 (24.8)	27 (24.8)	-	
**EI (kcal/day)**	776 (663, 951)	1340 (1118, 1605)	1795 (1612, 1998)	3250 (2503, 4221)	-	
**Age (years)**	11.8 (10.5, 13.2)	11.9 (10.6, 13.5)	11.8 (10.3, 13.4)	11.5 (10.4, 12.6)	0.616	N.S.
**BMI-percentile-for-age (%)**	66.7 (31.0, 144)	68.5 (40.0, 117)	55.8 (29.4, 106.1)	57.6 (22.5, 147)	0.665	N.S.
**Nutritional intake**						
Protein (% kcal/day)	12.9 (10.7, 15.6)	13.1 (11.0, 15.6)	12.1 (10.2, 14.3)	12.1 (10.2, 14.3)	0.223	N.S.
Fat (% kcal/day)	34.5 (30.2, 39.4)	34.8 (30.4, 39.8)	32.8 (27.0, 39.9)	34.1 (28.8, 40.4)	0.458	N.S.
Carbohydrate (% kcal/day)	53.2 (46.5, 60.9)	53.3 (47.9, 59.2)	56.2 (49.2, 64.3)	55.3 (49.2, 62.1)	0.274	N.S.
**Intake of confectionaries**						
Sweets and desserts (% energy)	11.5 (5.4, 23.4)	11.6 (4.6, 27.4)	13.7 (8.5, 21.9)	13.9 (7.4, 25.2)	0.605	N.S.
Sugary beverages (g/day)	95.5 (16.0, 545)	145 (25.6, 793)	245 (58.7, 1014)	557 (225, 1377)	< 0.001	**
Sugary beverages (g/EI)	0.15 (0.04, 0.49)	0.13 (0.04, 0.42)	0.16 (0.051, 0.44)	0.18 (0.08, 0.37)	0.819	N.S.

Variables were indicated as geometric mean (GM) (GM ± geometric standard deviation) except for the number of boys and girls n (%). EI/BMR: self-reported energy intake/ basal metabolic rate. First quartile: < 25^th^ percentile, second quartile: ≥ 25^th^ percentile and < 50^th^ percentile, third quartile: ≥ 50^th^ percentile and < 75^th^ percentile, and fourth quartile: ≥ 75^th^ percentile. N.S.: not significant

^a^One-way analysis of variance was used for assessing the associations of variables across each EI/BMR quartile (Bonferroni correction α = 0.05/8).

^¶^A significant differences (*P* < 0.05) as compare to quartiles with the third quartile by Dunnet t-test (α = 0.05).

**P < 0.001

To show the association between dietary habit, social support, and socio economic status and EI/BMR, Table 3 indicates the difference in age-adjusted mean EI/BMR by dietary habits, social support, and socioeconomic status using ANCOVA. In girls, the mean EI/BMR in the group of children who prepared food < 3.8 times/week (mean) was significantly lower than that of the group that did so ≥ 3.8 times/week (*P* = 0.030), but this was not observed in boys. There were significant differences between the frequency of breakfast in both the sexes (*P* = 0.021 in boys, *P* = 0.007 in girls). The Tukey’s test revealed that the mean EI/BMR in the group with frequency of breakfast 0–2 times/week in boys, and 3–4 times/week in girls, was significantly lower than the group that did so ≥ 5 times/week (*P* < 0.05). The items on social support score and socioeconomic status, such as caregiver’s education level and annual household income, did not show any significant associations with EI/BMR.

**Table 3:** The Differences in Age-Adjusted Mean EI/BMR by Dietary Habits, Social Support, and Socioeconomic Status.

	Boys				Girls			
	n (%)	aGM of EI/BMR (aGM ± GSE)	*P* ^a^		n (%)	aGM of EI/BMR (aGM ± GSE)	*P* ^a^	
**Frequency of food preparation**								
A household member (times/week)								
< mean (boys 5.6, girls 6.2)	57 (52.3)	0.98 (0.90, 2.9)	0.135	N.S.	54 (49.5)	1.1 (1.05, 1.2)	0.710	N.S.
≥ mean	52 (47.7)	1.2 (1.07, 3.5)			55 (50.5)	1.2 (1.09, 1.3)		
Children								
< mean (boys 3.5, girls 3.8)	56 (51.4)	1.02 (0.94, 3.0)	0.419	N.S.	53 (48.6)	1.02 (0.95, 1.1)	0.030	*
≥ mean	53 (48.6)	1.1 (1.03, 3.3)			56 (51.4)	1.3 (1.2, 1.4)		
**Frequency of breakfast (times/week)**								
0 − 2	19 (17.4)	0.75 (0.65, 2.4)^†^	0.021	*	27 (24.8)	1.06 (0.95, 1.2)	0.007	*
3 − 4	30 (27.5)	1.2 (1.05, 3.6)			32 (29.4)	0.93 (0.84, 1.03)^†^		
≥ 5	60 (55.0)	1.1 (1.05, 3.4)			50 (45.9)	1.4 (1.3, 1.5)		
**Social support score (units)**								
< 25^th^ percentile (boys 5.0, girls 6.0)	37 (34.0)	0.98 (0.89, 3.0)	0.340	N.S.	42 (38.5)	1.1 (1.03, 1.2)	0.735	N.S.
≥ 25^th^ percentile	72 (66.1)	1.1 (1.03, 3.3)			67 (61.5)	1.2 (1.09, 1.3)		
**Caregiver’s education level**								
Under high school level	58 (53.2)	1.09 (1.01, 3.2)	0.629	N.S.	76 (69.7)	1.2 (1.2, 1.3)	0.080	N.S.
Over college level	51 (46.8)	1.03 (0.95, 3.06)			33 (30.3)	1.0 (0.9, 1.1)		
**Annual household income (US dollars)**								
≤ 20,000	45 (41.3)	1.1 (1.01, 3.2)	0.625	N.S.	64 (58.7)	1.2 (1.09, 1.3)	0.810	N.S.
> 20,000 − ≤ 40,000	47 (43.1)	1.07 (0.98, 3.2)			35 (32.1)	1.1 (1.0, 1.2)		
> 40,000	17 (15.6)	0.94 (0.81, 3.0)			10 (9.1)	1.2 (1.03, 1.5)		

aGM (aGM ± GSE): age-adjusted geometric mean (aGM ± geometric standard error). EI/BMR: self-reported energy intake/ basal metabolic rate. N.S.: not significant.

^a^Analysis of covariance was used to compare the aGM of EI/BMR in the categories of dietary habits (frequency of food preparation by a household member and children and frequency of breakfast), social support score, and socioeconomic status (caregiver’s education level and annual household income).

^†^A significant difference (*P* < 0.05) in the aGM of EI/BMR as compared to the frequency of breakfast of ≥ 5 times/week using the Tukey’s test.

*P < 0.05

Table 4 presents the age-adjusted odds ratios of overweight and obesity in each quartile of EI/BMR, using the third quartile of EI/BMR as a control. As compared to the third quartile of EI/BMR, the lowest quartile showed a significantly higher prevalence of overweight [boys’ fully adjusted odds ratio (aOR) 7.3; 95% confidence interval (95% CI) 2.1, 30; *P* for trend < 0.001; girls’ aOR 5.3; 95% CI 1.6, 20; *P* for trend = 0.080] and higher prevalence of obesity (boys’ aOR 4.3; 95% CI 1.08, 20; *P* for trend = 0.005; girls’ aOR 4.1; 95% CI 1.02, 21; *P* for trend = 0.077). Additionally, in boys, the fourth quartile of EI/BMR showed a lower prevalence of overweight than that of the third quartile (aOR 0.22; 95% CI 0.047, 0.87).

**Table 4:** Adjusted Odds Ratio of Overweight and Obesity in the EI/BMR Quartiles as Compared to the Third Quartile by a Multiple Logistic Regression Model.

	EI/BMR									
	First quartile (under-reporting)(under-reporting)		Second quartile		Third quartile		Fourth quartile (over-reporting)(over-reporting)			
	Case/total (%))	aOR (95%CI)	Case/total (%)	aOR (95%CI)	Case/total (%)	Reference	Case/total (%)	aOR (95%CI)	*P* for trend	
**Boys**										
**Overweight**										
Model1	20/27 (74.1)	5.5 (1.8, 19)*	9/27 (33.3)	0.83 (0.26, 2.6)	10/28 (35.7)	1	4/27 (14.8)	0.26 (0.061, 0.96)^†^	<0.001	**
Model2		5.5 (1.8, 19)*		0.84 (0.26, 2.7)		1		0.26 (0.061, 0.96)^†^		
Model3		7.3 (2.1, 30)*		0.79 (0.22, 2.8)		1		0.22 (0.047, 0.87)*		
**Obesity**										
Model1	10/27 (37.0)	3.7 (1.03, 15)^¶^	6/27 (22.2)	1.6 (0.40, 7.1)	4/28 (14.3)	1	2/27 (7.4)	0.41 (0.053, 2.4)	0.005	*
Model2		3.8 (1.07, 16)*		1.7 (0.42, 7.7)		1		0.41 (0.052, 2.4)		
Model3		4.3 (1.08, 20)*		1.5 (0.302, 7.3)		1		0.29 (0.034, 1.8)		
**Girls**										
**Overweight**										
Model1	17/28 (60.7)	5.5 (1.7, 19)*	14/27 (51.9)	3.7 (1.2, 13)*	6/27 (22.2)	1	12/27 (44.4)	3.0 (0.93, 10)	0.080	N.S.
Model2		5.3 (1.7, 19)*		3.6 (1.1, 13)*		1		3.0 (0.93, 10)		
Model3		5.3 (1.6, 20)*		3.3 (0.96, 12)*		1		3.5 (0.99, 14)		
**Obesity**										
Model1	10/28 (35.7)	4.4 (1.2, 22)*	6/27 (22.2)	2.3 (0.53, 12)	3/27 (11.1)	1	5/27 (18.5)	1.8 (0.40, 9.7)	0.077	N.S.
Model2		4.6 (1.2, 23)*		2.3 (0.54, 12)		1		1.8 (0.40, 9.7)		
Model3		4.1 (1.02, 21)^§^		2.01 (0.43, 11)		1		1.5 (0.303, 8.1)		

EI/BMR: self-reported energy intake/ basal metabolic rate. aOR (95% CI): adjusted odds ratio, 95% confidence interval (25^th^ percentile, 75^th^ percentile). Model 1: The model was adjusted for age. Model 2: Model 1 + frequency of breakfast (times/week). Model 3: Model 2 + frequency of food preparation by children (times/week) + frequency of breakfast (times/week) + social support score (units) + caregiver’s education level (under high school level, Over college level) + annual household income (≤ 20,000, > 20,000 − ≤ 40,000, > 40,000). *P* for trend was analyzed by multiple logistic regression models for aOR of overweight and obesity. First quartile: < 25^th^ percentile, second quartile: ≥ 25^th^ percentile and < 50^th^ percentile, third quartile: ≥ 50^th^ percentile and < 75^th^ percentile, and fourth quartile: ≥ 75^th^ percentile. N.S.: not significant

*P < 0.05,

**P < 0.001,

^†^*P* = 0.052,

^¶^*P* = 0.055,

^§^*P* = 0.061

## Discussion

### Summary of the main result

We found that the lower EI than BMR was associated with the prevalence of overweight and obesity among AA children. Moreover, the lower ratio of EI/BMR was associated with higher BMI-percentile-for-age in boys. We confirmed there was no selection bias through comparing the main result analyzed including ineligible children (total n = 283; boys n = 131, girls n = 152) and excluding them (data not shown). We excluded ineligible participants in order to be able to compare each result among the same number of participants.

### Availability of prediction of basal metabolic rate and food frequency questionnaire

A previous study showed that the mean of BMR measured by body calorimetric data was 1,298 kcal/day (SD = 108) among 42 AA girls aged 13.5 years (SD = 1.7) [20]. The mean of BMR by the doubly labeled water was 1,147 kcal/day (SD = 239) among the subjects aged 7–12 years and 1,601 kcal/day (SD = 359) in those aged 13–17 years [21]. The mean of estimated BMR in the present study was similar to this report.

Another study showed that the average energy intake, as determined through the *Block Kids FFQ*, was 1,245 kcal/day (SD = 569) among children aged less than 12 years and 1,801 kcal/day (SD = 1,010) among children aged more than 12 years (53% girls and 21% AA of the total) [11]. The inter-class correlations (ICC) of the *Block Kids FFQ* for the reliability were 0.63 with energy intake; 0.21, 0.35, and 0.39 with energy from protein, fat, and carbohydrate, respectively among 18 children aged 10 to 17 years [11]. The Pearson correlation for the validity between the *Block Kids FFQ* and 24-hour dietary recall were ranged from 0.45–0.68 for energy intake, 0.72–0.78, 0.18–0.67, 0.42–0.91 for percent energy form protein, fat, and carbohydrate, respectively among 75–83 children aged 10 to 17 years [11]. Another study showed that the Spearman correlation coefficient (weighted kappa statistics) of soda pop, which were included as one of the confectionaries in the FFQ, was 0.223 to 0.326 between three-day diary and *Block Kids FFQ* [22]. The validity and the reliability are generally acceptable to use as a self-reported consumption as compared to the other FFQ among children [23].

### Under- and over-reporting of energy intake and body composition

It has been said that EI/BMR ranged 1.35–1.55 is a conservative estimation for the normally active population to maintain body weight beyond metabolic costs [24, 25]. According to the cutoff, the third quartile of EI/BMR (≥ 1.00 and < 1.68 in boys, ≥ 1.13 and < 1.64 in girls) could assess reasonable values for the estimation of actual food intake in the present study. While, boys in the fourth quartile were leaner than boys in the third quartile in spite of overconsumption (>1.55 EI/BMR). It may be appropriate to consider that the energy expenditure of the fourth quartile could be higher than their energy consumption. Another possibility is that leaner boys in the fourth quartile overestimated their food intake as compared to their BMI-percentile-for-age.

Some studies have reported the association of under-reporting of energy intake and body mass index with overweight and obesity in children [26–29]. Overweight and obese males and females aged 13−16 years had a higher tendency to under-report their energy intake than were normal-weight individuals. This was assessed using the ratio of EI calculated using the Food Behavior Questionnaire (combined with 24h dietary recall, FFQ, and other items) and EI/BMR [26]. One report also indicated that AA girls aged 8–10 years, who underestimated their food intake calculated from 2 days of recall data, showed higher BMI, which was measured using (EI/BMR)/estimated minimum energy requirement [27]. Hence, our findings show consistency of the results in AA children from the *BHCK* project.

The present study showed that the absolute amount of consumption of confectionaries were significantly lower in all other quartiles as compared to that in the third quartile, although the percentages of EI had no differences across the quartiles. This may indicate the omission of less-healthy items, such as sweets, desserts, and sugary beverages among children, who generally had a tendency to eat more sweet snacks [30]. This attitude may be induced by a degree of systematic social desirability bias regarding body shape and fear of being judged based on their body shape and dietary habits. Additionally, a previous report inferred that school children have access to food from home, friends, and local shops between meals, and the frequency of consuming snacks plays a role in unstructured eating patterns [31, 32]. If the participants ate snacks frequently, they might not be able to accurately remember and report their snack consumption. Thus, under-reporting among 10–14-year-old children in the current study might be affected by complex factors such as social desirability and levels of snacking.

### Dietary habit and the self-reported energy intake

We hypothesized that frequent preparation of food in a household would enable children to develop dietary awareness and behavior through food intention and nutrition knowledge [1, 2, 33]. It has been indicated that food preparation by parents or children results in positive motivation for self-control and interest in healthy food choice among children [34]. The frequency of food preparation by children may have an effect on proper estimation of food intake especially for girls in the present study.

We found that frequency of breakfast might be one of the confounding factors regarding the under-reporting of food intake. Previous reports showed that “concern for health” and “daily routine” were influenced by food choice for breakfast in adolescents [35]. Therefore, we hypothesized that children who ate breakfast frequently may be able to report their food intake more accurately, which was supported by our results.

### Sex- and age- difference of the self-reported energy intake

Some of our findings were inconsistent with previous reports. Studies have reported the proportion of underreporting food intake was higher in girls than in boys [28, 29]. There are also reports that girls may be more affected by the social desirability of good health [29]. In contrast, direction of girls, boys had a conflicting result between overconsumption (>1.55 EI/BMR) despite lower BMI-percentile-for-age in the fourth quartile of EI/BMR. It is possible that girls in the fourth quartile may estimate their food intake appropriately regarding their BMI-percentile-for-age compared to boys in the fourth quartile. In addition, boys prepared foods by themselves less frequently than girls did. According to these results, girls may have more awareness of their dietary habits than boys, in addition to, or being affected social desirability, as suggested by other studies. Some studies indicated that older children tended to under-report their food intake [24, 25, 36]. However, the present study did not show any significant differences in age across the quartiles. The result indicates older age did not necessarily associate with underreporting of food intake.

### Social support and socioeconomic status and the self-reported energy intake

Social support score, caregiver’s education level, and annual household income did not show significant associations with EI/BMR. A previous study inferred that socioeconomic status influenced the reliability of self-reported food intake [31]. However, previous studies reported that socio-demographic factors, such as caregiver’s education level or household income, were not significantly association with the under-reported energy intake among AA girls and adolescent in Canada [25, 28]. The results of the present remain inconclusive regarding the influence of caregiver’s education level and household income on awareness of food intake. A long-term intervention studies are needed to confirm these factors and misreporting of food intake.

### Strengths and limitations

The strengths of our study are that the data were collected from both children and their adult caregivers. This enabled us to obtain the information children could not provide, such as caregiver’s education level and household income. In addition, this research was conducted in an important population that this highly impacted by overweight and obesity and could clearly benefit from increased understanding of dietary intakes and habits.

However, the present study also has some limitations. First, we did not measure actual BMR using the doubly labeled water method [21, 37]. Instead of the measured BMR, we calculated predicted BMR using evidence based equations. In addition, it was difficult to assess if the reason for the inconsistency between BMI-percentile-for-age in the fourth quartile and the mean EI/BMR in boys was overestimating against BMR or higher energy expenditure compared to EI. This was because we did not measure the level of energy expenditure such as physical activity. We made this decision because the primary outcomes and intervention strategies of the parent *BHCK* study were diet-related, and we chose not to collect physical activity data to reduce participant burden. Second, a limitation of the data collection is that we did not record percentages of body fat, and did not measure waist circumference. Nonetheless, body mass index has been confirmed for use in adolescent screening programs to predict subjects with excess body fat [38, 39]. Third, it is possible that the FFQ could not cover all the variety of foods the participants consumed. However, the *Block Kids FFQ* is widely used to investigate dietary habits of children in epidemiological studies [11–13]. Fourth, the frequency of food preparation, caregiver’s education level, and household income were self-reported by a caregiver. Fifth, we could not infer causality between awareness of food intake and BMI in this cross-sectional study. Furthermore, we should assess obesity not only with BMI-percentile-for-age but also abdominal measurement and body fat for finding central obesity. Although there were some limitations, based on previous reports that under-reporting of energy intake may predict overweight and obesity, we confirmed that the results of the current study were rational.

### Conclusion

In conclusion, we identified the possibility that low-income, urban, AA children who under-reported EI against BMR had a high prevalence of overweight and obesity. The results from our study imply that frequent food preparation by children and eating breakfast may help children develop the cognition of their food intake. If children have the capability of monitoring their dietary habits and health interests, it could help them attain a healthy weight, stabilize the weight of those at risk of being overweight, or even decrease the number of overweight children. Nevertheless, before we assess the self-reported food intake of children, it is necessary to note that overweight and obese children may report their food intake less than their actual food intake for some complex reasons. Based on our findings, researchers and practitioners should be cautious in interpreting self-reported energy intake alone especially among overweight/obese children. Rather multiple measures would be most appropriate to assess self-reported energy intake.

## Supporting Information

S1 Table Sex Differences in the Baseline Demographics of the Study Participants. (DOCX)
