# Supplementary material for: Inconsistency between Self-Reported Energy Intake and Body Mass Index among Urban, African-American Children
Source: PLoS One. 2016 Dec 15;11(12):e0168303. doi: 10.1371/journal.pone.0168303 (PMC5158042; doi:10.1371/journal.pone.0168303)
Supplement: S1 Table — (DOCX) [file pone.0168303.s001.docx]

**S1 Table. Sex Differences in the Baseline Demographics of the Study Participants.**

|  | **Boys** | |  | **Girls** | |  |  |
| --- | --- | --- | --- | --- | --- | --- | --- |
|  | **n (%)** | **GM (GM ± GSD)** |  | **n (%)** | **GM (GM ± GSD)** | ***P*** |  |
| **Age (years)** |  |  |  |  |  |  |  |
| 10-11 | 46 (42.2) |  |  | 53 (48.6) |  | 0.616 | N.S. |
| 12-13 | 43 (39.5) |  |  | 37 (34.0) |  |  |  |
| 14 | 20 (18.5) |  |  | 19 (17.4) |  |  |  |
| **Body composition** |  |  |  |  |  |  |  |
| Body weight (kg) | 109 | 51.0 (36.9, 70.3) |  | 109 | 51.9 (37.6, 71.6) | 0.690 | N.S. |
| BMI-percentile-for-age (%) | 109 | 59.4 (33.6, 105) |  | 109 | 61.9 (29.8, 129) | 0.643 | N.S. |
| Overweight | 43 (39.5) |  |  | 49 (45.0) |  | 0.411 | N.S. |
| Obesity | 22 (20.2) |  |  | 24 (22.0) |  | 0.740 | N.S. |
| **Energy- intake and - expenditure** |  |  |  |  |  |  |  |
| Self-reported energy intake: EI (kcal/day) | 109 | 1656 (953, 2879) |  | 109 | 1559 (894, 2719) | 0.423 | N.S. |
| Basal metabolic rate: BMR (kcal_th_) | 109 | 1557 (1294, 1873) |  | 109 | 1350 (1195, 1526) | <0.001 | ** |
| EI/BMR | 109 | 1.06 (0.58, 1.96) |  | 109 | 1.2 (0.65, 2.05) | 0.310 | N.S. |
| **Dietary habit** |  |  |  |  |  |  |  |
| Frequency of food preparation (times/week) |  |  |  |  |  |  |  |
| A household member | 109 | 5.6 (2.4, 12.0) |  | 109 | 6.2 (2.7, 12.8) | 0.369 | N.S. |
| Children | 109 | 3.5 (0.9, 9.8) |  | 109 | 3.8 (1.0, 10.1) | 0.658 | N.S. |
| Frequency of breakfast (times/week) | 109 | 3.7 (3.1, 7.09) |  | 109 | 3.2 (2.6, 6.7) | 0.074 | N.S. |
| 0 − 2 | 19 (17.4) |  |  | 27 (24.8) |  | 0.307 | N.S. |
| 3 − 4 | 30 (27.5) |  |  | 23 (29.4) |  |  |  |
| ≥ 5 | 60 (55.1) |  |  | 59 (45.9) |  |  |  |
| **Social support** |  |  |  |  |  |  |  |
| Social-support score (units)^a^ | 109 | 6.0 (5.0, 7.0) |  | 109 | 7.0 (6.0, 7.0) | 0.006 | * |
| **Socioeconomic status** |  |  |  |  |  |  |  |
| Caregiver’s education level |  |  |  |  |  |  |  |
| Under high school level | 58 (53.2) |  |  | 76 (69.7) |  | 0.012 | * |
| Over college level | 51 (46.8) |  |  | 33 (30.3) |  |  |  |
| Annual household income |  |  |  |  |  |  |  |
| ≤ 20,000 (US dollars) | 45 (41.3) |  |  | 64 (58.7) |  | 0.032 | * |
| > 20,000 − ≤ 40,000 | 47 (43.1) |  |  | 35 (32.1) |  |  |  |
| > 40,000 | 17 (15.6) |  |  | 10 (9.2) |  |  |  |

Sex difference was assessed by student t-test (continuous variables), Wilcoxon rank-sum test (social-support scores), and chi-square test (categorical variables).

^a^The result of social-support score indicated median (25^th^ percentile, 75^th^ percentile).

GM: geometric mean, the exponentiated value of the log-transformed mean.

GSD: geometric standard deviation, the exponentiated value of the standard deviation of the log-transformed value.

N.S.: not significant

**P* < 0.05, ***P* < 0.001

**Table 2. The Differences in Age, BMI-Percentile-For-Age and Dietary Intake Across the Quartiles of EI/BMR.**

|  | **EI/BMR** | | | | | | |  |  |  |
| --- | --- | --- | --- | --- | --- | --- | --- | --- | --- | --- |
|  | **First quartile (under-reporting)** |  | **Second quartile** |  | **Third quartile** |  | **Fourth quartile (over-reporting)** |  | ***P*^a^** |  |
| **Boys: EI/BMR** | **< 0.65** |  | **≥ 0.65 and <1.00** |  | **≥ 1.00 and < 1.68** |  | **≥ 1.68** |  |  |  |
| **n (%)** | 27 (24.8) |  | 27 (24.8) |  | 28 (25.7) |  | 27 (25.7) |  | - |  |
| **EI (kcal/day)** | 882 (729, 1069) |  | 1256 (1030, 1533) |  | 2051 (1606, 2619) |  | 3283 (2374, 4540) |  | - |  |
| **Age (years)** | 12.2 (10.9, 13.7) |  | 11.7 (10.4, 13.1) |  | 12.1 (10.8, 13.6) |  | 11.3 (10.1, 12.8) |  | 0.094 | N.S. |
| **BMI-percentile-for-age (%)** | 85.2 (70.1, 104) ^¶^ |  | 55.3 (31.0, 98.7) |  | 54.2 (26.9, 109) |  | 49.0 (28.6, 83.9) |  | 0.001 | ** |
| **Nutritional intake** |  |  |  |  |  |  |  |  |  |  |
| Protein (% energy) | 13.3 (11.0, 16.1) |  | 13.0 (10.5, 16.0) |  | 13.0 (11.1, 15.2) |  | 13.0 (11.3, 14.9) |  | 0.955 | N.S. |
| Fat (% energy) | 32.6 (26.8, 39.5) |  | 32.1 (27.7, 37.2) |  | 32.9 (28.9, 37.4) |  | 34.9 (30.3, 40.1) |  | 0.223 | N.S. |
| Carbohydrate (% energy) | 54.6 (47.5, 62.7) |  | 55.5 (50.1, 61.5) |  | 55.1 (49.6, 61.6) |  | 53.3 (47.5, 59.9) |  | 0.621 | N.S. |
| **Intake of confectionaries** |  |  |  |  |  |  |  |  |  |  |
| Sweets and desserts (% energy) | 11.9 (4.6, 28.6) |  | 14.6 (7.8, 26.8) |  | 13.7 (7.7, 23.7) |  | 13.9 (8.0, 23.5) |  | 0.708 | N.S. |
| Sugary beverages (g/day) | 74.1 (9.8, 520) ^¶^ |  | 152 (28.6, 795) |  | 294 (109, 793) |  | 405 (107, 1521) |  | < 0.001 | ** |
| Sugary beverages (g/EI) | 0.11 (0.03, 0.41) |  | 0.15 (0.04, 0.44) |  | 0.15 (0.06, 0.34) |  | 0.14 (0.06, 0.30) |  | 0.783 | N.S. |
| **Girls: EI/BMR** | **< 0.74** |  | **≥ 0.74 and <1.13** |  | **≥ 1.13 and < 1.64** |  | **≥ 1.64** |  |  |  |
| **n (%)** | 28 (25.7) |  | 27 (24.8) |  | 27 (24.8) |  | 27 (24.8) |  | - |  |
| **EI (kcal/day)** | 776 (663, 951) |  | 1340 (1118, 1605) |  | 1795 (1612, 1998) |  | 3250 (2503, 4221) |  | - |  |
| **Age (years)** | 11.8 (10.5, 13.2) |  | 11.9 (10.6, 13.5) |  | 11.8 (10.3, 13.4) |  | 11.5 (10.4, 12.6) |  | 0.616 | N.S. |
| **BMI-percentile-for-age (%)** | 66.7 (31.0, 144) |  | 68.5 (40.0, 117) |  | 55.8 (29.4, 106.1) |  | 57.6 (22.5, 147) |  | 0.665 | N.S. |
| **Nutritional intake** |  |  |  |  |  |  |  |  |  |  |
| Protein (% kcal/day) | 12.9 (10.7, 15.6) |  | 13.1 (11.0, 15.6) |  | 12.1 (10.2, 14.3) |  | 12.1 (10.2, 14.3) |  | 0.223 | N.S. |
| Fat (% kcal/day) | 34.5 (30.2, 39.4) |  | 34.8 (30.4, 39.8) |  | 32.8 (27.0, 39.9) |  | 34.1 (28.8, 40.4) |  | 0.458 | N.S. |
| Carbohydrate (% kcal/day) | 53.2 (46.5, 60.9) |  | 53.3 (47.9, 59.2) |  | 56.2 (49.2, 64.3) |  | 55.3 (49.2, 62.1) |  | 0.274 | N.S. |
| **Intake of confectionaries** |  |  |  |  |  |  |  |  |  |  |
| Sweets and desserts (% energy) | 11.5 (5.4, 23.4) |  | 11.6 (4.6, 27.4) |  | 13.7 (8.5, 21.9) |  | 13.9 (7.4, 25.2) |  | 0.605 | N.S. |
| Sugary beverages (g/day) | 95.5 (16.0, 545) |  | 145 (25.6, 793) |  | 245 (58.7, 1014) |  | 557 (225, 1377) |  | < 0.001 | ** |
| Sugary beverages (g/EI) | 0.15 (0.04, 0.49) |  | 0.13 (0.04, 0.42) |  | 0.16 (0.051, 0.44) |  | 0.18 (0.08, 0.37) |  | 0.819 | N.S. |

Variables were indicated as geometric mean (GM) (GM ± geometric standard deviation) except for the number of boys and girls n (%).

EI/BMR: self-reported energy intake/ basal metabolic rate

^a^One-way analysis of variance was used for assessing the associations of variables across each EI/BMR quartile (Bonferroni correction α = 0.05/8).

First quartile: < 25^th^ percentile, second quartile: ≥ 25^th^ percentile and < 50^th^ percentile, third quartile: ≥ 50^th^ percentile and < 75^th^ percentile, and fourth quartile: ≥ 75^th^ percentile

^¶^A significant differences (*P* < 0.05) as compare to quartiles with the third quartile by Dunnet t-test (α = 0.05).

N.S.: not significant

***P* < 0.001

**Table 3. The Differences in Age-Adjusted Mean EI/BMR by Dietary Habits, Social Support, and Socioeconomic Status.**

|  | **Boys** | | | |  | **Girls** | | | |
| --- | --- | --- | --- | --- | --- | --- | --- | --- | --- |
|  | **n (%)** | **aGM of EI/BMR (aGM ± GSE)** | ***P* ^a^** |  |  | **n (%)** | **aGM of EI/BMR (aGM ± GSE)** | ***P* ^a^** |  |
| **Frequency of food preparation** |  |  |  |  |  |  |  |  |  |
| A household member (times/week) |  |  |  |  |  |  |  |  |  |
| < mean (boys 5.6, girls 6.2) | 57 (52.3) | 0.98 (0.90, 2.9) | 0.135 | N.S. |  | 54 (49.5) | 1.1 (1.05, 1.2) | 0.710 | N.S. |
| ≥ mean | 52 (47.7) | 1.2 (1.07, 3.5) |  |  |  | 55 (50.5) | 1.2 (1.09, 1.3) |  |  |
| Children |  |  |  |  |  |  |  |  |  |
| < mean (boys 3.5, girls 3.8) | 56 (51.4) | 1.02 (0.94, 3.0) | 0.419 | N.S. |  | 53 (48.6) | 1.02 (0.95, 1.1) | 0.030 | * |
| ≥ mean | 53 (48.6) | 1.1 (1.03, 3.3) |  |  |  | 56 (51.4) | 1.3 (1.2, 1.4) |  |  |
| **Frequency of breakfast (times/week)** |  |  |  |  |  |  |  |  |  |
| 0 − 2 | 19 (17.4) | 0.75 (0.65, 2.4)^†^ | 0.021 | * |  | 27 (24.8) | 1.06 (0.95, 1.2) | 0.007 | * |
| 3 − 4 | 30 (27.5) | 1.2 (1.05, 3.6) |  |  |  | 32 (29.4) | 0.93 (0.84, 1.03)^†^ |  |  |
| ≥ 5 | 60 (55.0) | 1.1 (1.05, 3.4) |  |  |  | 50 (45.9) | 1.4 (1.3, 1.5) |  |  |
| **Social support score (units)** |  |  |  |  |  |  |  |  |  |
| < 25^th^ percentile (boys 5.0, girls 6.0) | 37 (34.0) | 0.98 (0.89, 3.0) | 0.340 | N.S. |  | 42 (38.5) | 1.1 (1.03, 1.2) | 0.735 | N.S. |
| ≥ 25^th^ percentile | 72 (66.1) | 1.1 (1.03, 3.3) |  |  |  | 67 (61.5) | 1.2 (1.09, 1.3) |  |  |
| **Caregiver’s education level** |  |  |  |  |  |  |  |  |  |
| Under high school level | 58 (53.2) | 1.09 (1.01, 3.2) | 0.629 | N.S. |  | 76 (69.7) | 1.2 (1.2, 1.3) | 0.080 | N.S. |
| Over college level | 51 (46.8) | 1.03 (0.95, 3.06) |  |  |  | 33 (30.3) | 1.0 (0.9, 1.1) |  |  |
| **Annual household income (US dollars)** |  |  |  |  |  |  |  |  |  |
| ≤ 20,000 | 45 (41.3) | 1.1 (1.01, 3.2) | 0.625 | N.S. |  | 64 (58.7) | 1.2 (1.09, 1.3) | 0.810 | N.S. |
| > 20,000 − ≤ 40,000 | 47 (43.1) | 1.07 (0.98, 3.2) |  |  |  | 35 (32.1) | 1.1 (1.0, 1.2) |  |  |
| > 40,000 | 17 (15.6) | 0.94 (0.81, 3.0) |  |  |  | 10 (9.1) | 1.2 (1.03, 1.5) |  |  |

aGM (aGM ± GSE): age-adjusted geometric mean (aGM ± geometric standard error)

EI/BMR: self-reported energy intake/ basal metabolic rate

^a^Analysis of covariance was used to compare the aGM of EI/BMR in the categories of dietary habits (frequency of food preparation by a household member and children and frequency of breakfast), social support score, and socioeconomic status (caregiver’s education level and annual household income).

^†^A significant difference (*P* < 0.05) in the aGM of EI/BMR as compared to the frequency of breakfast of ≥ 5 times/week using the Tukey’s test.

N.S.: not significant

**P* < 0.05

**Table 4. Adjusted Odds Ratio of Overweight and Obesity in the EI/BMR Quartiles as Compared to the Third Quartile by a Multiple Logistic Regression Model.**

|  | **EI/BMR** | | | | | | | | | | |  |  |  |
| --- | --- | --- | --- | --- | --- | --- | --- | --- | --- | --- | --- | --- | --- | --- |
|  | **First quartile (under-reporting)** | |  | **Second quartile** | |  | **Third quartile** | |  | **Fourth quartile (over-reporting)** | |  |  |  |
|  | **Case/total (%)** | **aOR (95%CI)** |  | **Case/total (%)** | **aOR (95%CI)** |  | **Case/total (%)** | **Reference** |  | **Case/total (%)** | **aOR (95%CI)** |  | ***P* for trend** |  |
| **Boys** |  |  |  |  |  |  |  |  |  |  |  |  |  |  |
| **Overweight** |  |  |  |  |  |  |  |  |  |  |  |  |  |  |
| Model1 | 20/27 (74.1) | 5.5 (1.8, 19)* |  | 9/27 (33.3) | 0.83 (0.26, 2.6) |  | 10/28 (35.7) | 1 |  | 4/27 (14.8) | 0.26 (0.061, 0.96)^†^ |  | <0.001 | ** |
| Model2 |  | 5.5 (1.8, 19)* |  |  | 0.84 (0.26, 2.7) |  |  | 1 |  |  | 0.26 (0.061, 0.96)^†^ |  |  |  |
| Model3 |  | 7.3 (2.1, 30)* |  |  | 0.79 (0.22, 2.8) |  |  | 1 |  |  | 0.22 (0.047, 0.87)* |  |  |  |
| **Obesity** |  |  |  |  |  |  |  |  |  |  |  |  |  |  |
| Model1 | 10/27 (37.0) | 3.7 (1.03, 15)^¶^ |  | 6/27 (22.2) | 1.6 (0.40, 7.1) |  | 4/28 (14.3) | 1 |  | 2/27 (7.4) | 0.41 (0.053, 2.4) |  | 0.005 | * |
| Model2 |  | 3.8 (1.07, 16)* |  |  | 1.7 (0.42, 7.7) |  |  | 1 |  |  | 0.41 (0.052, 2.4) |  |  |  |
| Model3 |  | 4.3 (1.08, 20)* |  |  | 1.5 (0.302, 7.3) |  |  | 1 |  |  | 0.29 (0.034, 1.8) |  |  |  |
| **Girls** |  |  |  |  |  |  |  |  |  |  |  |  |  |  |
| **Overweight** |  |  |  |  |  |  |  |  |  |  |  |  |  |  |
| Model1 | 17/28 (60.7) | 5.5 (1.7, 19)* |  | 14/27 (51.9) | 3.7 (1.2, 13)* |  | 6/27 (22.2) | 1 |  | 12/27 (44.4) | 3.0 (0.93, 10) |  | 0.080 | N.S. |
| Model2 |  | 5.3 (1.7, 19)* |  |  | 3.6 (1.1, 13)* |  |  | 1 |  |  | 3.0 (0.93, 10) |  |  |  |
| Model3 |  | 5.3 (1.6, 20)* |  |  | 3.3 (0.96, 12)* |  |  | 1 |  |  | 3.5 (0.99, 14) |  |  |  |
| **Obesity** |  |  |  |  |  |  |  |  |  |  |  |  |  |  |
| Model1 | 10/28 (35.7) | 4.4 (1.2, 22)* |  | 6/27 (22.2) | 2.3 (0.53, 12) |  | 3/27 (11.1) | 1 |  | 5/27 (18.5) | 1.8 (0.40, 9.7) |  | 0.077 | N.S. |
| Model2 |  | 4.6 (1.2, 23)* |  |  | 2.3 (0.54, 12) |  |  | 1 |  |  | 1.8 (0.40, 9.7) |  |  |  |
| Model3 |  | 4.1 (1.02, 21)^§^ |  |  | 2.01 (0.43, 11) |  |  | 1 |  |  | 1.5 (0.303, 8.1) |  |  |  |

EI/BMR: self-reported energy intake/ basal metabolic rate

aOR (95% CI): adjusted odds ratio, 95% confidence interval (25^th^ percentile, 75^th^ percentile)

Model 1: The model was adjusted for age.

Model 2: Model 1 + frequency of breakfast (times/week).

Model 3: Model 2 + frequency of food preparation by children (times/week) + frequency of breakfast (times/week) + social support score (units) + caregiver’s education level (under high school level, Over college level) + annual household income (≤ 20,000, > 20,000 − ≤ 40,000, > 40,000).

*P* for trend was analyzed by multiple logistic regression models for aOR of overweight and obesity.

First quartile: < 25^th^ percentile, second quartile: ≥ 25^th^ percentile and < 50^th^ percentile, third quartile: ≥ 50^th^ percentile and < 75^th^ percentile, and fourth quartile: ≥ 75^th^ percentile

N.S.: not significant

**P* < 0.05, ***P* < 0.001, ^†^*P* = 0.052, ^¶^*P* = 0.055, ^§^*P* = 0.061
